# Heparin-binding Hemagglutinin of *Mycobacterium tuberculosis* Is an Inhibitor of Autophagy

**DOI:** 10.3389/fcimb.2017.00033

**Published:** 2017-02-07

**Authors:** Qing Zheng, Zhi Li, Shan Zhou, Qian Zhang, Lei Zhou, Xiaorui Fu, Liu Yang, Yueyun Ma, Xiaoke Hao

**Affiliations:** ^1^Department of Clinical Laboratory, Xijing Hospital, Fourth Military Medical UniversityXi'an, China; ^2^Department of Pharmacology, School of Pharmacy, Fourth Military Medical UniversityXi'an, China

**Keywords:** HBHA, A549, *Mycobacterium tuberculosis*, *Mycobacterium smegmatis*, autophagy, apoptosis

## Abstract

Airway epithelial cell is often the initial site of attack by pathogens, and cell death is commonly caused by internalization of *Mycobacterium tuberculosis* (*Mtb*). However, the mechanism of interaction between epithelial cells and *Mtb* is not well understood. In this study, we investigated the role of the heparin-binding hemagglutinin (HBHA) protein of *Mtb* in the function of epithelial cells. In particular, the autophagy of A549 cells was determined based on microtubule-associated protein 1 light chain 3 alpha (LC3) activity. Autophagosome formation was detected by Monodansylcadaverine (MDC) staining and immune fluorescence staining of LC3. Autophagy could be significantly suppressed by HBHA protein. In addition, the LDH assay results showed that HBHA treatment could induce death on A549 cells. To explore the form of cell death, we detected the activity of caspase-3 and LDH release of A549 cells in the presence or absence of caspase inhibitor Z-VAD-FMK. Results demonstrated that HBHA treatment could induce apoptosis of A549 cells. To further confirm these results, we constructed the recombinant *Mycobacterium smegmatis* (*MS*) expressing HBHA (*rMS-HBHA*) and explored the influence of *rMS-HBHA* on the function of A549 cells. *rMS-HBHA* infection significantly inhibited LC3 expression and the maturation of autophagosomes in A549 cells. Subsequently, we infected A549 cells with *MS* and detected the viability of intracellular *MS* by CFU counts. *rMS-HBHA* showed higher survival and replication capacity in A549 cells than those of the wild-type *MS*. Finally, infection of A549 cells with *rMS-HBHA* caused further apoptosis. These findings suggested that *rMS-HBHA* could inhibit autophagy, promote its survival and replication within A549 cells, and subsequently induce apoptosis on infected cells to facilitate infection.

## Introduction

Tuberculosis (TB) remains a devastating disease with approximately 2 billion people infected worldwide and 1.2 million deaths in 2010 (Hawn et al., 2015). Approximately 36 million people will die from TB annually by 2020 (Moliva et al., 2015). The etiological agent of TB, that is, *Mycobacterium tuberculosis* (*Mtb*), is the most successful intracellular pathogen that can invade and replicate in many host cell types, including both phagocytic and nonphagocytic cells (Vir et al., 2014).

Alveolar epithelium is often the initial site of the lung's response against *Mtb*. It is composed of type I and type II pneumocytes, which form a cell layer that provides a barrier function (Lin et al., 1998; Chuquimia et al., 2013). Increasing evidence implicated that alveolar epithelium, particularly type II pneumocyte, plays an important role in both host cell defense and bacterial dissemination (Xiong et al., 2014; Fine-Coulson et al., 2015; Ryndak et al., 2015). *Mycobacterium smegmatis* (*MS*) takes advantage of macropinocytosis for entry into epithelial cells, and internalized *MS* are killed by A549 cells (Garcia-Perez et al., 2008). However, unlike *MS*, type II pneumocytes could provide a permissive position for Mtb to replicate considerably and ultimately help bacterial dissemination (Bermudez and Goodman, 1996; Ryndak et al., 2015). The mechanism of how *Mtb* destroys the defense system is still unknown.

Autophagy is an intracellular self-digestion process whereby cytoplasmic constituents are delivered to and degraded by lysosomes (Lamb et al., 2013). Upon sensing stress conditions, such as starvation, MTOR is inhibited, which is required to activate the ULK complex. The Beclin-1 complex is activated by sensing the activation of the ULK complex. Subsequently, ATG12–ATG5 and LC3II are generated, and the membrane is elongated to form a double-membraned vesicle, that is, the autophagosome. Finally, the autophagosome fuses with lysosome, thereby forming an autolysosome to digest the cargo (Chen et al., 2014). Autophagy is crucial for quality control, energy supply, and immune defense against invading bacterial and viral pathogens. This process can eliminate intracellular pathogens through inflammation regulation, antigen presentation, and microorganism capture and degradation (Deretic et al., 2013; Lamb et al., 2013). Autophagy could also kill intracellular pathogens, such as *Mtb*, on the basis of strong degradative and other antimicrobial properties distinct to autolysosomes (Bradfute et al., 2013). Our previous studies using transmission electron microscope (TEM) revealed that *Mtb* bacilli-containing compartments are surrounded with double membranes, which characterize the autophagic process in A549. In addition, induction of autophagy in A549 presents a protective role against *Mtb* infection. *Mtb* could cause further necrosis among LC3-silenced A549 than that among wild-type A549 (Guo et al., 2013). Therefore, autophagy maybe the main mechanism that defends against invasion of pathogen (Li et al., 2012; Thurston et al., 2012; Wileman, 2013). Although autophagy is an efficient mechanism for clearing pathogens, such as *Mtb*, the mechanisms by which *Mtb* avoid being killed by autophagy remain unknown. Identifying and understanding the role of *Mtb* proteins that are critical to this process are considerably significant and will help us understand whether host cell autophagy or *Mtb* proteins can be targeted by new therapeutics.

Heparin-binding hemagglutinin (HBHA), a major adhesin in *Mtb* (Esposito et al., 2011; Lebrun et al., 2012), is involved in the attachment of mycobacteria to epithelial cells and plays vital role in the dissemination of *Mycobacterium* from the site of primary infection (Locht et al., 2006; Esposito et al., 2011). Thus, *Mycobacterium* may use HBHA to inhibit autophagy and thereby facilitate infection. To confirm this hypothesis, we explored the role of the *Mtb* protein HBHA in regulating autophagy in host airway epithelial cells using the A549 cell line. To further validate our results, we used the fast-growing, nonpathogenic *MS MC*_*2*_*155* strain (Snapper et al., 1990), which lacks the *hbhA* gene, to construct a recombinant *MS* strain that expressed HBHA (*rMS-HBHA*) (Delogu et al., 2004). Our results demonstrated that *rMS-HBHA* could inhibit autophagy, promote its survival and replication within A549 cells, and subsequently induce apoptosis of infected cells to facilitate infection.

## Materials and methods

### Cells and culture

The human non-small-cell lung carcinoma A549 cell line was obtained from The Cell Bank of the Chinese Academy of Sciences (Shanghai, China). The cells were grown in modified RPMI-1640 medium (HyClone, USA) supplemented with 10% heat-inactivated fetal bovine serum (Gibco, New Zealand) at 37°C in a humidified incubator with a 5% CO_*2*_ atmosphere.

### Bacteria strains

The wild-type strain of *M. smegmatis MC*_*2*_*155* strain was obtained from the Department of Clinical Laboratory, Xijing Hospital, Fourth Military Medical University. Cells were grown in 7H9/7H10 Middlebrook (BD, USA) broth supplemented with 0.05% Tween 80, OADC (BD, USA), and 0.2% glycerol (v/v). Cells were grown at 37°C with continuous agitation (220 rpm).

### Expression and purification of HBHA purified protein

To produce recombinant HBHA (rHBHA) in *Escherichia coli*, the corresponding genes were PCR amplified using *M. tuberculosis* H37Rv (ATCC27294) DNA as the template and the following oligonucleotide primers: F primer (5′-CACGGATCCATGGCTGAAAACTCGAACAT-3′) and R primer (5′-CTGAAGCTTACTACTTCTGGGTGACCTTC-3′). The forward and reverse primers contained the underlined BamHI and HindIII restriction sites, respectively. The PCR products were digested with BamHI and HindIII and cloned into the PQE80L vector (Laboratory Animal Center, The Fourth Military Medical University), and the resulting clones were sequenced (Sangon Biotech). *E. coli* strain BL21 (DE3), which expresses His-tagged protein, was grown in Luria–Bertani broth supplemented with 100 μg/ml ampicillin. After induction with 0.3 mM isopropy-β-D-thiogalactoside for 5 h, the cells were lysed by sonication for 30 min. The rEC-HBHA was purified by heparin–Sepharose chromatography and further purified using a His-GraviTrap purification kit (GE Healthcare, USA) in accordance with the manufacturer's instructions.

### Construction of recombinant *rMS-HBHA* and *rMS-HBHA-GFP*

The fast-growing, nonpathogenic *M. smegmatis MC*_*2*_*155* strain (Snapper et al., 1990), which lacks the *hbhA* gene, was electroporated to obtain a mycobacteria recombinant strain that could be easily manipulated in most laboratories (Delogu et al., 2004). The shuttle plasmid vector pMV261 or pMN437, which contains GFP promoter and the full-length *hbhA* gene, was transformed through electroporation and grown in 7H9 broth containing kanamycin (15 μg/ml) and hygromycin B (20 μg/ml). Such vector was obtained from Dr. Babak Javid of the Medical College of Tsinghua University. *E*. *coli* strain BL21 (Takara, Japan) was used for transformation and protein expression and cultivated under standard conditions. The purified protein of HBHA was prepared by our laboratory. The *hbhA* gene was excised using the enzymes BamHI and HindIII and cloned at the same site of the mycobacterial shuttle vector pMV261. Alternatively, the *hbhA* gene was excised using the enzymes ClaI and HindIII, cloned at the same site of the pMN437 vector, and electroporated (2.5 kV, 25 μF, and 1000 Ω) for the preparation of *M. smegmatis* competent cells using standard techniques (Bardarov et al., 1997).

### Monodansylcadaverine (MDC) staining of autophagic vacuoles

To analyze the formation of autophagosome, A549 cells were starved for 90 min and subsequently treated with HBHA (8 μg/ml) for 90 min or 3-MA (100 μg/ml), an inhibitor of autophagy, for 4 h. For the infection assay, A549 cells were infected with *MS-GFP* and *rMS-HBHA-GFP* at the multiplicity of infection (MOI) of 10:1 for 18 h. Afterward, the A549 cells were washed three times with PBS and treated with 50 μM MDC in an incubator for 15 min. The cells were again washed three times with PBS and then immediately observed under a laser confocal microscope (FV10i, Olympus, Tokyo, Japan).

### Immune fluorescence detection of LC3

The A549 cells were cultured in glass bottom cell culture dish (NEST, Hong Kong, China) and starved for 90 min. Afterward, A549 cells were treated with HBHA (8 μg/ml) for 90 min or 3-MA (100 μg/ml) for 4 h. The cells were washed three times with PBS and fixed with 4% paraformaldehyde for 15 min at room temperature. Cells were treated with 0.3% Triton-100 20 min at room temperature to increase permeability. Subsequently, they were blocked with heat-inactivated fetal bovine serum (Gibco, New Zealand) for 30 min at room temperature. The cells were incubated with polyclonal antiLC3 antibody at 4°C overnight. After washing with PBS, cells were incubated with antirabbit Cy3 fluorescent secondary antibody (1:500 final dilution; BBI Life Sciences, China), and they were observed by laser confocal microscopy (FV10i, Olympus, Tokyo, Japan).

### Western blot analysis

To detect the expression of the key proteins of autophagy, A549 cells were starved for 90 min and treated with HBHA of different concentrations for 90 min or with rapamycin or rapamycin plus 3-MA (100 μg/ml) for 4 h. To confirm the effect of HBHA on the autophagic flux of A549 cells, cells were treated with 350 nM bafilomycin A1 (BAF A1; Abcam, USA) to prevent lysosomal degradation. For caspase-3 detection, A549 cells were treated with HBHA for 18 h in the presence or absence of caspase inhibitor Z-VAD-FMK (20 μM). The cells were then lysed in cell lysis buffer with phenylmethylsulfonyl fluoride, phosphatase inhibitor, and protease inhibitor according to the instructions of the manufacturer of the protein extraction kit. The protein concentration in the lysate was quantified using a BCA protein assay kit. Equal amounts of protein from each sample were separated by SDS-PAGE and transferred to polyvinylidene fluoride membranes (EMD Millipore, Billerica, MA, USA). Subsequent to incubation in blocking buffer (LICOR, Odyssey, USA) for 1 h, the membranes were incubated with monoclonal primary antibodies against ATG5/LC3B/Beclin-1 (Abcam, USA)/Caspase-3 (GeneTex, USA) HBHA (antiHBHA was prepared by our laboratory) and β-actin (Abcam, USA) overnight at 4°C. The membranes were then incubated with a horseradish peroxidase-conjugated goat antirabbit IgG secondary antibody (LICOR, Odyssey, USA) or antimouse IgG secondary antibody (LICOR, Odyssey, USA) for 2 h. The bands were detected using a dual-color infrared laser (LICOR, Odyssey, USA), and the protein levels were quantitated by densitometry using Gel-Pro Analyzer software (Media Cybernetics, Inc., Rockville, MD, USA).

### LDH detection by enzyme labeling

The A549 cells were seeded at a density of 1 × 10^6^ cells/well in six-well plates and incubated with HBHA of different concentrations for 24 h in the presence or absence of rapamycin or 3-MA. Alternatively, A549 cells were infected with *MS* or *rMS-HBHA* at the MOI of 10:1 for 24 h. Afterward, the culture supernatant was collected and added to a 96-well plate. The caspase inhibitor Z-VAD-FKM (20 μM) was added into cells 1 h prior to HBHA treatment or infection. Specific RIPK1 inhibitor Necrostatin-1 (Nec-1) (Sigma) was used at 30 μM. A LDH cytotoxicity kit was then used according to the manufacturer's instructions. Specifically, dinitrophenylhydrazine and sodium hydroxide solution were detected at 450 nm using a microplate reader.

### Detection of bacterial CFU in A549 cells infected with *MS* and *rMS*

For the assay, A549 cells were seeded onto 24-well plates at a density of 4 × 10^5^ cells/well. The cells were infected with *MS* or *rMS-HBHA* at the MOI of 10:1 for 3 h in the presence or absence of rapamycin or 3-MA at 37°C in 5% CO_2_. For caspase inhibition, Z-VAD-FKM was added into cells 1 h prior to infection. Amikacin (200 mg/ml) was then added for 3 h to kill extracellular bacteria. At indicated time points, the cells were washed three times with basal RPMI 1640 medium, and viable intracellular bacteria were released by incubation with 0.5 ml of 0.1% Triton X-100 in sterile water for 10 min. The samples were mixed vigorously with 0.5 ml of 7H9 broth. Serial 10-fold dilutions of lysates were prepared in 7H9 broth and plated on 7H10 agar for determination of CFU numbers.

### Statistical analysis

All experiments were repeated at least three times. Statistical analyses were performed using SPSS 17.0. Data are expressed as the mean ± standard deviation and were analyzed using one-way ANOVA. *P* < 0.05 was considered to indicate a statistically significant difference.

## Results

### HBHA inhibited LC3 and Beclin-1 expression in A549 cells

**Figure 1:**
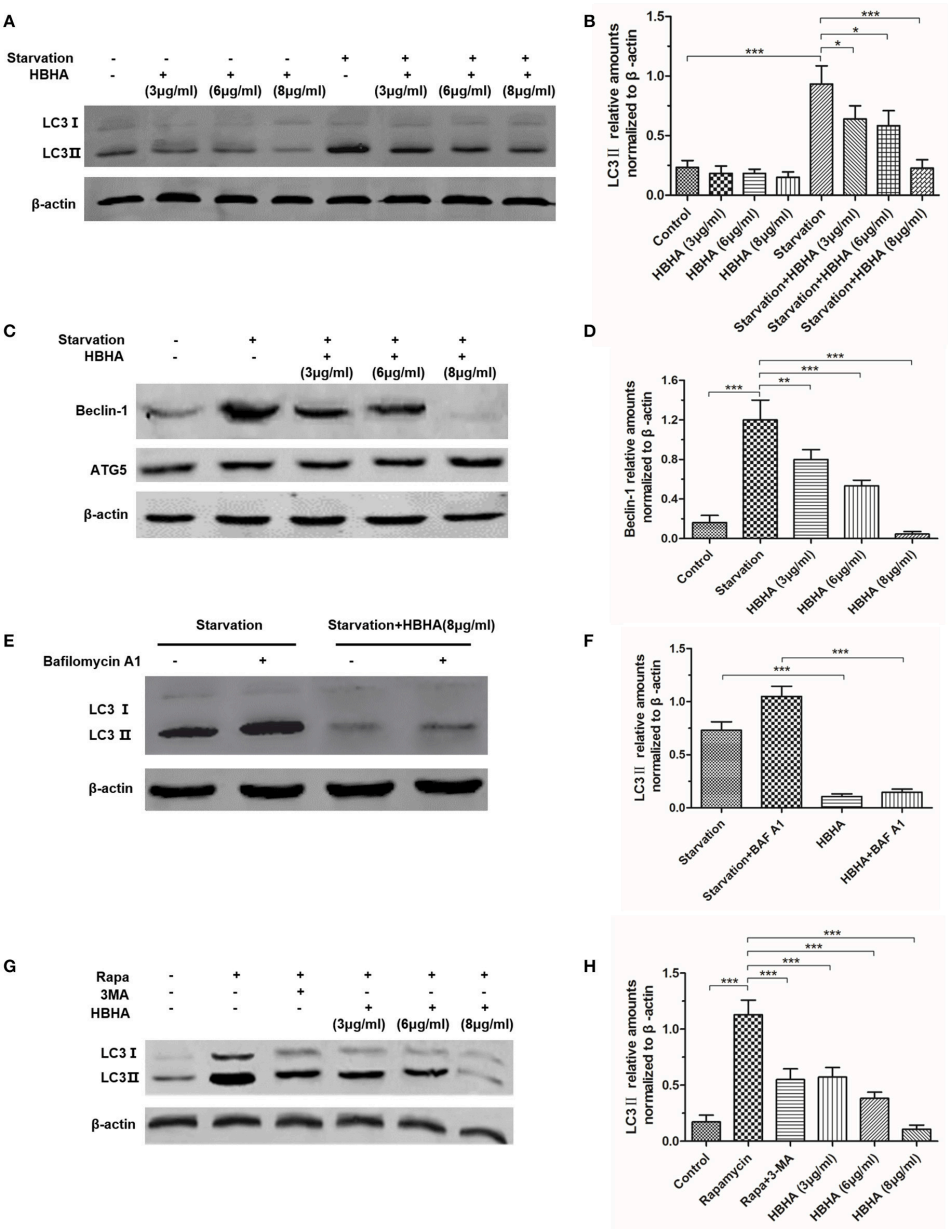
**Heparin-binding hemagglutinin (HBHA) inhibited the expression of LC3 and Beclin-1 in A549 cells. (A–D)** A549 cells were starved for 1 h, and HBHA proteins of different concentrations were subsequently added to the cells for 90 min. LC3 **(A)** and ATG5/Beclin-1 **(C)** expression was detected by Western blot. The intensities of LC3II **(B)** and Beclin-1 **(D)** bands were normalized to the intensity of β-actin. (^*^P < 0.05, ^**^P < 0.01, ^***^P < 0.001 vs. starvation group in one-way ANOVA, *n* = 3). **(E)** To measure the effect of HBHA on the autophagic flux of A549 cells, the cells were treated with bafilomycin A1 (BAF A1) to prevent lysosomal degradation. LC3 expression was detected by Western blot. **(F)** The intensities of LC3II bands were normalized to the intensity of β-actin. (^***^P < 0.001 in one-way ANOVA, *n* = 3). **(G)** A549 cells were treated with rapamycin alone or rapamycin plus 3-MA or HBHA of different concentrations. Afterward, LC3 expression was detected by Western blot. **(H)** The intensities of LC3II bands were normalized to the intensity of β-actin. (^***^P < 0.001 vs. rapamycin group in one-way ANOVA, *n* = 3).

To evaluate the effect of HBHA on the autophagy of A549 cells, Western blot assay was performed to detect the expression of LC3 and Beclin-1. LC3 and Beclin-1, which were starved for 1 h, were significantly induced in A549. However, when HBHA was added after 90 min, the expression of LC3 and Beclin-1 was suppressed in a dose-dependent manner (Figures 1A–D). In particular, 8 μg/ml HBHA could remarkably inhibit the expression of starved LC3 and Beclin-1, and the ATG5 expression was not affected by HBHA (Figure 1C). Subsequently, to measure the effect of HBHA on the autophagic flux of A549 cells, the cells were treated with BAF A1 to prevent lysosomal degradation. Compared with BAF A1 alone, HBHA treatment with BAF A1 could decrease LC3II levels. This result confirmed that HBHA inhibited the synthesis of autophagy-related membranes (Figures 1E,F). When rapamycin or 3-MA was used to induce or inhibit autophagy, the LC3 expression could be suppressed by 3-MA. Similar to 3-MA, the LC3 expression was inhibited in a dose-dependent manner when HBHA was added (Figures 1G,H).

### HBHA inhibited the formation of autophagosome in A549 cells

**Figure 2:**
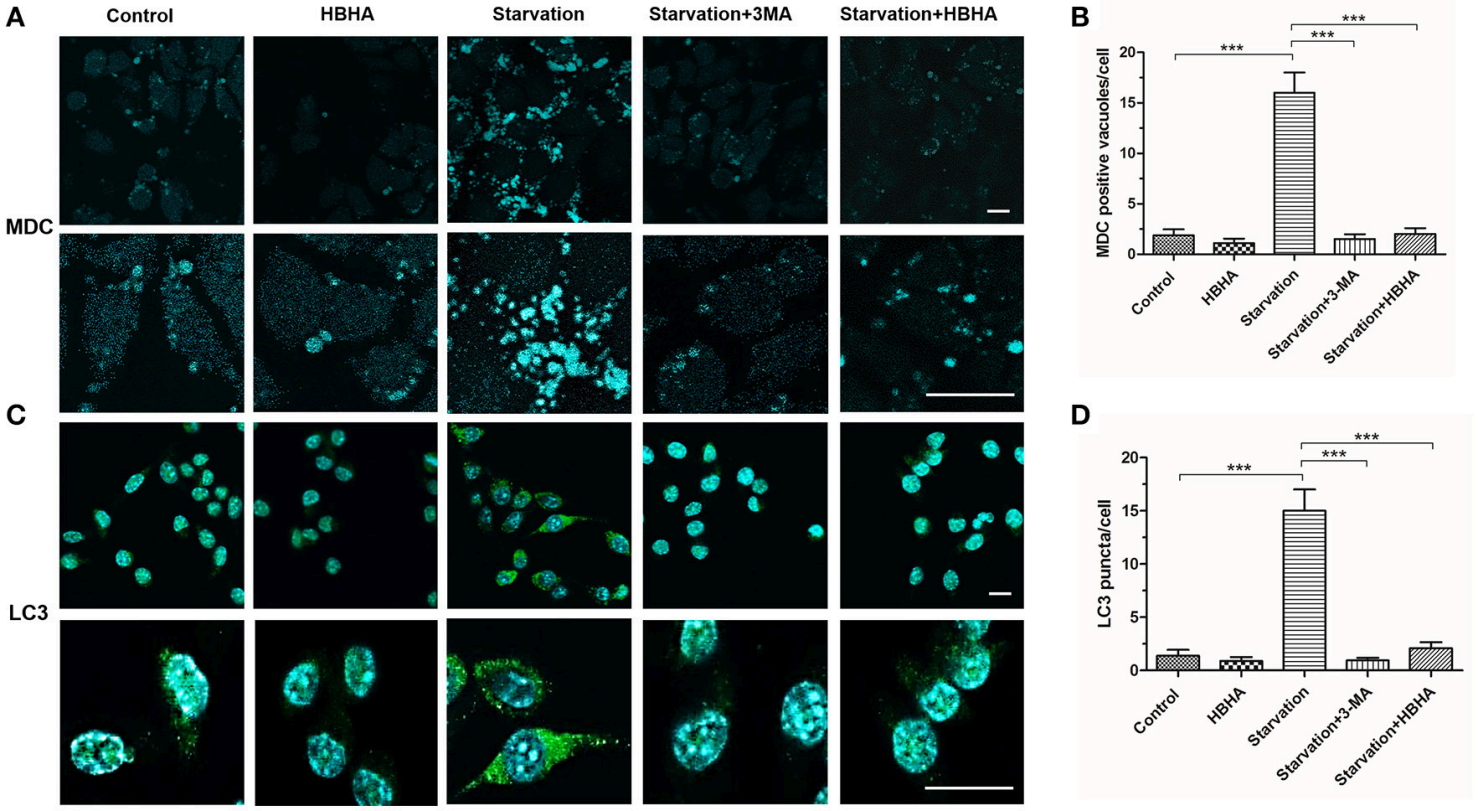
**HBHA inhibited the maturation of autophagosome in A549 cells. (A)** A549 cells were starved for 1 h and subsequently treated with HBHA (8 μg/ml) for 90 min or 3-MA (100 μg/ml) for 4 h. The cells were then stained with monodansylcadaverine (MDC), and MDC-labeled autophagic vacuoles were detected by confocal microscopy. Scale bars: 20 μm. **(B)** MDC-positive autophagic vacuoles were quantified in each group. (^***^P < 0.001 vs. starvation group in one-way ANOVA). **(C)** Immune fluorescence of LC3 puncta in A549 cells was detected by confocal microscopy. Scale bars: 20 μm. **(D)** LC3 puncta were quantified in each group. (^***^P < 0.001 vs. starvation group in one-way ANOVA).

To further explore the effect of HBHA on autophagy, we observed the formation of autophagosomes using MDC staining and immunofluorescence staining of LC3 in A549 cells. The number of MDC foci increased after starvation, but it could be reversed by 3-MA. Similarly, HBHA decreased the number of MDC foci (Figures 2A,B), and the number of LC3 puncta decreased significantly after HBHA addition (Figures 2C,D). Taken together, these results demonstrated that autophagy maturation could be considerably suppressed by HBHA.

### *rMS-HBHA* inhibited autophagy among starved A549 cells

**Figure 3:**
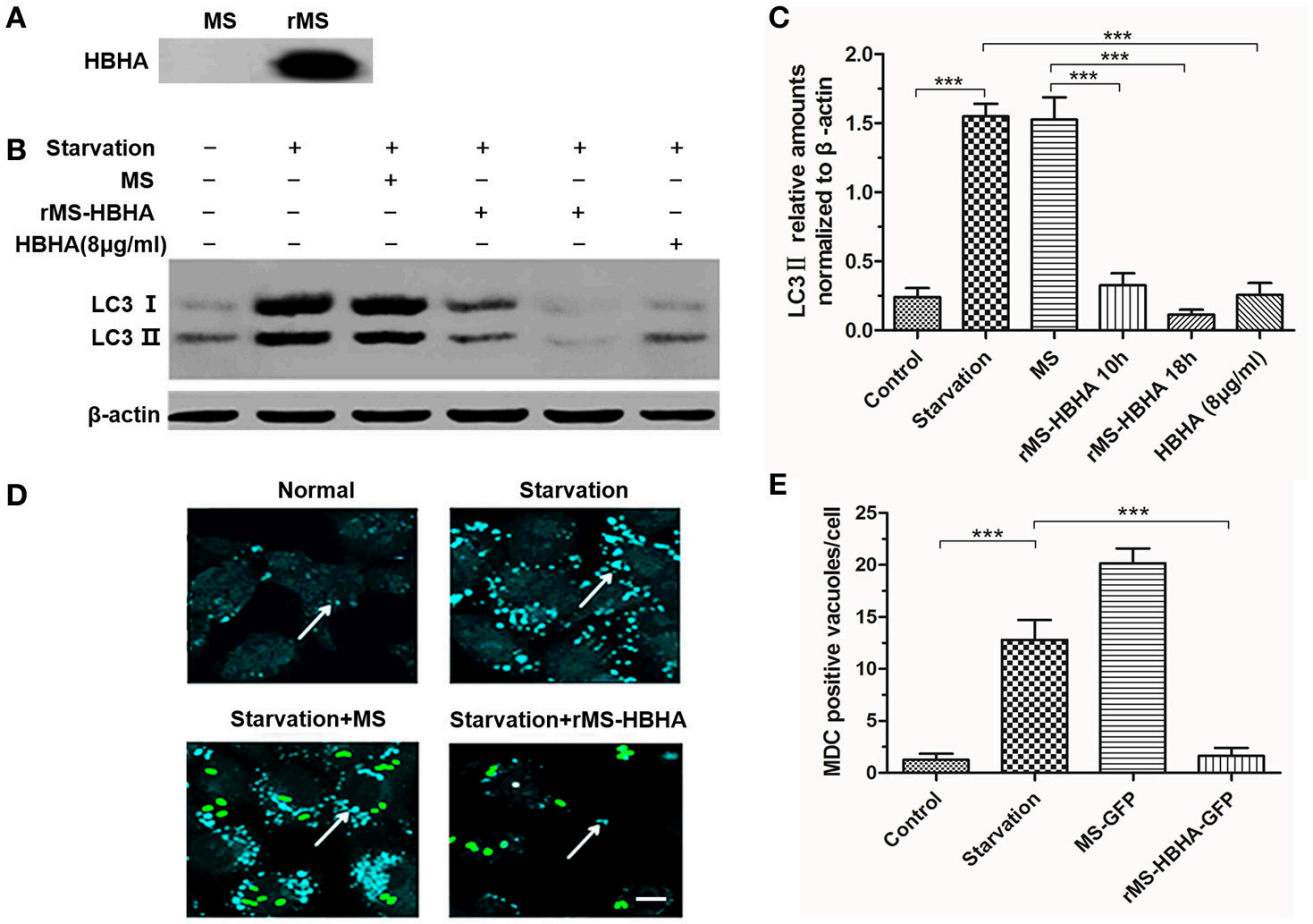
**Recombinant ***Mycobacterium smegmatis*** (***MS***) expressing HBHA (***rMS-HBHA***) inhibited autophagy among starved A549 cells. (A)** Total proteins of *MC*_*2*_*155* and *rMS-HBHA* were extracted, and Western blot was carried out to detect HBHA using anti-HBHA antibody. **(B)** A549 cells were infected with the wild-type strain *MC*_*2*_*155* or *rMS-HBHA* at the multiplicity of infection (MOI) of 10:1 for indicated time or treated with HBHA (8 μg/ml) for 90 min. The expression levels of LC3 were detected by Western blot. **(C)** The intensities of LC3II bands were normalized to the intensity of β-actin. (^***^P < 0.001 in one-way ANOVA, *n* = 3). **(D)** A549 cells were infected with GFP-expressing strains, and autophagosomes were stained with MDC. The white arrows indicate autophagosomes. **(E)** MDC-positive autophagic vacuoles were quantified in each group. (^***^P < 0.001 vs. starvation group in one-way ANOVA).

We transferred HBHA into *MS MC*_*2*_*155* to construct recombinant *rMS-HBHA*. To confirm that HBHA was successfully expressed in *rMS-HBHA*, the total proteins of *MS* and *rMS-HBHA* were extracted. Western blot analysis was performed to detect the expression of HBHA protein using anti-HBHA antibody. The results showed that HBHA was not expressed in the parent strain *MS MC*_*2*_*155*, but expressed in the *rMS-HBHA* (Figure 3A). To further confirm the inhibition role of HBHA on autophagy, A549 cells were infected with *MS* or *rMS-HBHA*. Western blot analysis results showed that *rMS-HBHA* inhibited the expression of starvation-induced LC3, but the wild-type strain did not (Figures 3B,C). We constructed GFP-expressing *MS* and *rMS-HBHA*, infected A549, and performed MDC staining to examine autophagosome formation. The number of MDC foci increased after starvation, and *rMS-HBHA* significantly decreased the number of MDC foci (Figures 3D,E). These results suggested that *rMS-HBHA* inhibited the maturation of autophagy and consistent with those observed with pure HBHA protein.

### Inhibition of autophagy could promote survival of *rMS-HBHA* within A549 cells

**Figure 4:**
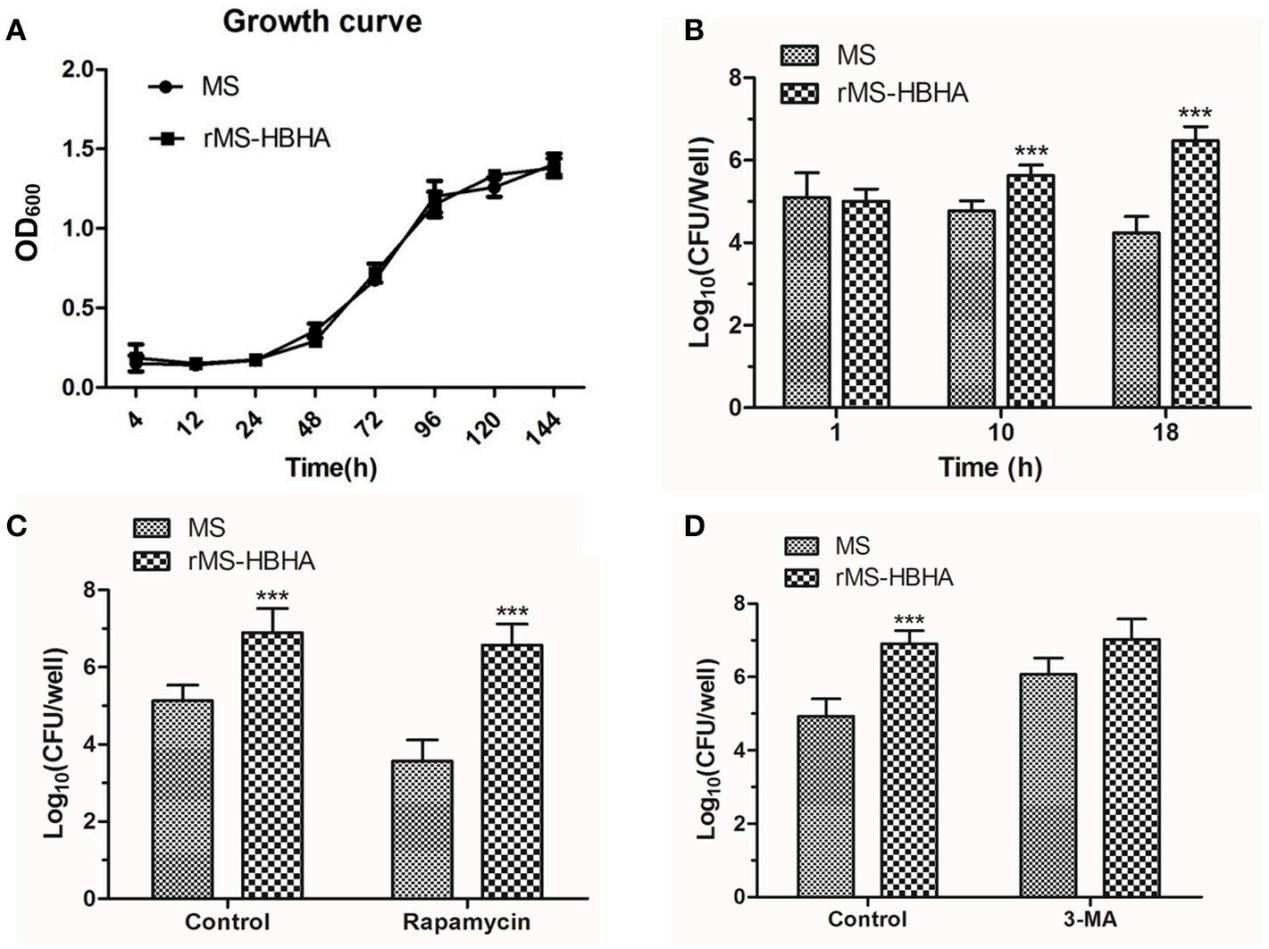
**Inhibition of autophagy could promote survival of ***rMS-HBHA*** within A549 cells. (A)** Growth curves of *MS* and *rMS-HBHA* were measured by OD_600_ absorbance. **(B)** A549 cells were infected with *MS* or *rMS-HBHA* at the MOI of 10:1. The cells were lysed at 1, 10, or 18 h post-infection, and lysates were diluted and plated on agar plates to determine the number of viable intracellular bacteria. (^***^P < 0.001 in two-way ANOVA, *n* = 3) **(C)** A549 cells were infected with *MS* or *rMS-HBHA* at the MOI of 10:1 and treated with rapamycin. Viable intracellular bacteria were measured at 18 h post-infection. (^***^P < 0.001 in one-way ANOVA, *n* = 3) **(D)** A549 cells were infected with *MS* or *rMS-HBHA* at the MOI of 10:1 and treated with 3-MA. Viable intracellular bacteria were measured at 18 h post-infection. (^***^P < 0.001 in one-way ANOVA, *n* = 3).

To determine whether autophagy inhibition could promote survival of *rMS-HBHA* within A549 cells, A549 cells were infected with WT *MS* or *rMS-HBHA* at the MOI of 10:1. Cells were lysed at 1, 10, or 18 h post-infection, and lysates were diluted and plated on agar plates to determine the number of viable intracellular bacteria. *MS* and *rMS-HBHA* showed no significant difference in growth curve (Figure 4A). However, *rMS-HBHA* showed higher survival and replication capacity within A549 cells than those of wild-type *MS* (Figure 4B). Autophagy activation in *MS*-infected A549 cells by rapamycin could decrease the number of intracellular *MS*, but showed no significant effect on *rMS-HBHA* (Figure 4C). Moreover, autophagy inhibition in *MS*-infected A549 cells by 3-MA could increase the number of intracellular *MS* (Figure 4D). These findings suggested that HBHA may enhance the capacity of *MS* to infect and survive within A549 cells through inhibition of autophagy.

### HBHA protein and *rMS-HBHA* infection could induce cell death of A549

**Figure 5:**
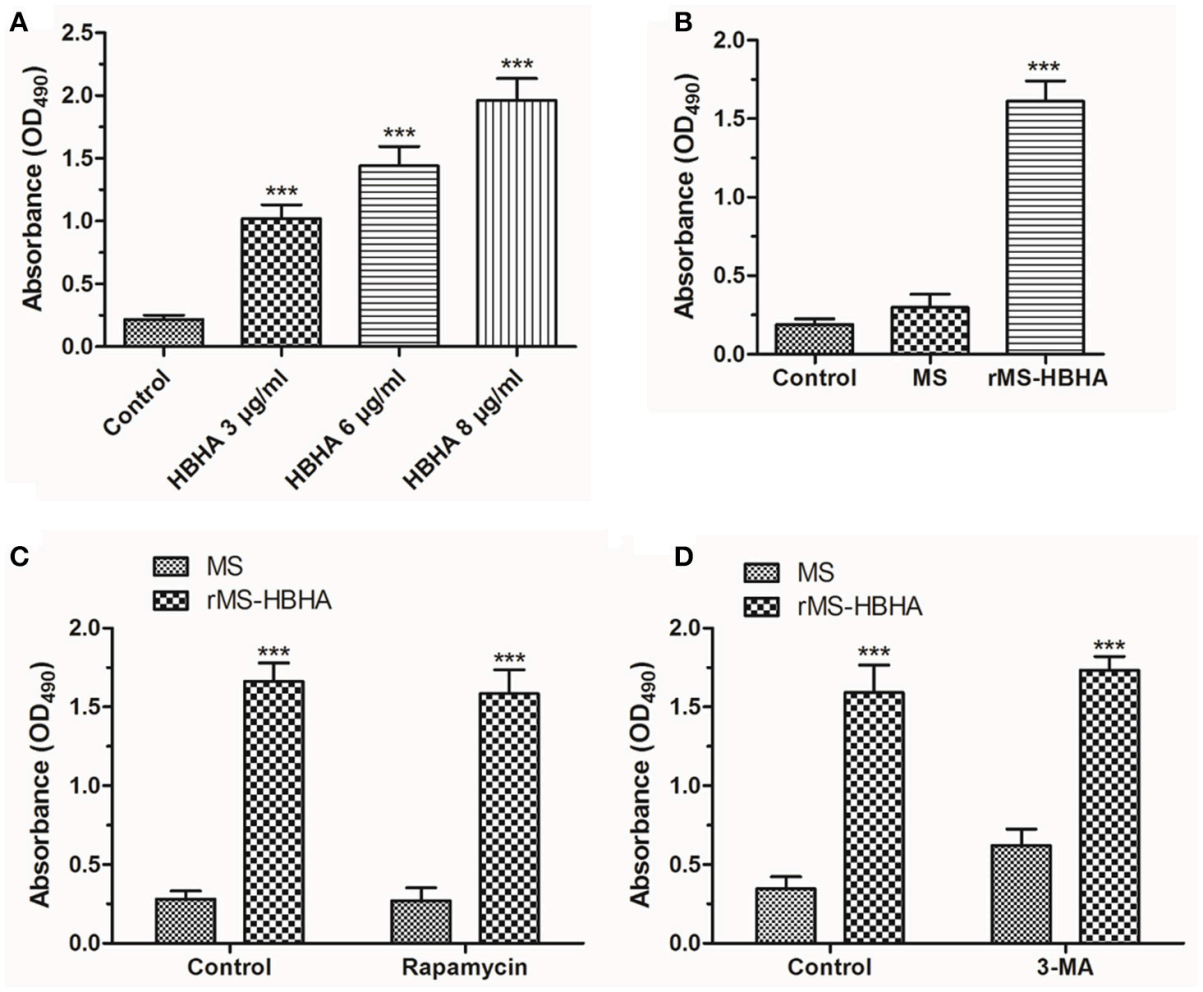
**HBHA protein and ***rMS-HBHA*** infection could induce cell death on A549. (A)** A549 cells were treated with HBHA of different concentrations for 24 h, and LDH release was detected. (^***^P < 0.001 vs. control group in one-way ANOVA, *n* = 3). **(B)** A549 cells were infected with MS or *rMS-HBHA* at the MOI of 10:1 for 24 h, and LDH release was detected. (^***^P < 0.001 vs. control group in one-way ANOVA, *n* = 3). **(C)** A549 cells were infected with MS or *rMS-HBHA* and treated with rapamycin, and LDH release was detected. (^***^P < 0.001 in one-way ANOVA, *n* = 3) **(D)** A549 cells were infected with MS or *rMS-HBHA* and treated with 3-MA, and LDH release was detected. (^***^P < 0.001 in one-way ANOVA, *n* = 3).

To determine the fate of infected A549 cells, A549 cells were treated with HBHA of different concentrations or infected with *MS* or *rMS-HBHA* at the MOI of 10:1 for 24 h. The culture supernatant was collected to measure the released LDH. We found that HBHA protein treatment could induce large amount of cell death (Figure 5A). Similarly, *rMS-HBHA* infection could induce great number of cell death but not wild-type *MS* (Figure 5B). Activation of autophagy in *rMS-HBHA*-infected A549 cells by rapamycin showed no effect on cell death (Figure 5C). Furthermore, inhibition of autophagy in *MS*-infected A549 cells by 3-MA could slightly increase cell death but showed no effect on *rMS-HBHA*-infected A549 cells (Figure 5D). These results suggested that HBHA could promote cell death in infected A549 cells.

### HBHA treatment induced apoptosis of A549 cells through activation of caspase-3

**Figure 6:**
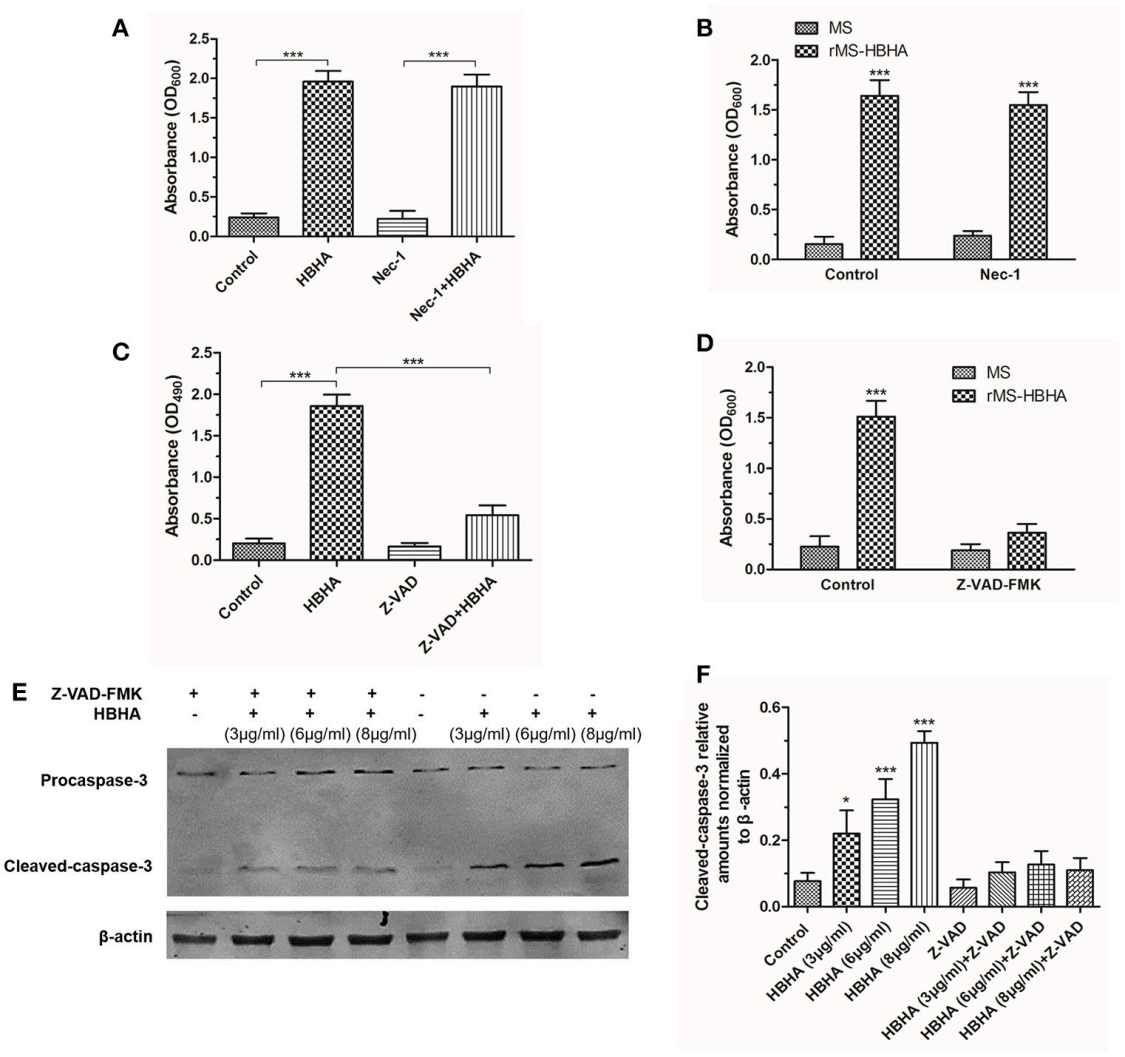
**HBHA treatment induced apoptosis on A549 cells through activation of caspase-3. (A)** A549 cells were treated with HBHA (8 μg/ml) for 24 h in the presence or absence of RIPK1 inhibitor Nec-1 (30 μM), and LDH release was detected. (^***^P < 0.001 in one-way ANOVA, *n* = 3). **(B)** A549 cells were infected with *MS* or *rMS-HBHA* in the presence or absence of Nec-1, and LDH release was detected. (^***^P < 0.001 in one-way ANOVA, *n* = 3) **(C)** A549 cells were treated with HBHA (8 μg/ml) for 24 h in the presence or absence of caspase inhibitor Z-VAD-FMK (20 μM), and LDH release was detected. (^***^P < 0.001 in one-way ANOVA, *n* = 3). **(D)** A549 cells were infected with *MS* or *rMS-HBHA* in the presence or absence of Z-VAD-FMK, and LDH release was detected. (^***^P < 0.001 in one-way ANOVA, *n* = 3) **(E)** A549 cells were treated with HBHA at different concentrations for 18 h in the presence or absence of Z-VAD-FMK. Caspase-3 expression was detected by Western blot. **(F)** The intensities of cleaved-caspase-3 bands were normalized to the intensity of β-actin. (^*^P < 0.05, ^***^P < 0.001 vs. starvation group in one-way ANOVA, *n* = 3).

To investigate the form of HBHA-induced cell death, the RIPK1 inhibitor Nec-1 and caspase inhibitor Z-VAD-FMK was used. HBHA- or *rMS-HBHA* infection-induced cell death was not affected after Nec-1 treatment (Figures 6A,B). These results suggested necroptosis was not involved in this process. However, HBHA- or *rMS-HBHA* infection-induced cell death was significantly attenuated after Z-VAD-FMK treatment (Figures 6C,D). This result suggested that apoptosis may participate in this process. To further confirm the results, the cleaved-caspase-3 activity was detected by Western blot. HBHA treatment could increase the expression of cleaved-caspase-3 in a dose-dependent manner, and treatment with Z-VAD-FMK could inhibit the activation of caspase-3 (Figures 6E,F). These results indicated that HBHA protein treatment or *rMS-HBHA* infection could induce apoptosis of A549 cells through activation of caspase-3.

## Discussion

Among most of the *Mtb* virulence factors, HBHA is particularly important in the infection process and *Mtb* immune evasion (Sechi et al., 2006). The amount of HBHA protein in alveolar type II epithelial cells increases considerably at 6 h post-infection, and the bacteria break through the cells after 8 h (de Lima et al., 2009), thereby indicating that HBHA facilitates the dispersion and replication of *Mtb* in lung epithelial cells. By contrast, HBHA-deficient *Mtb* mutant strains are significantly impaired in their ability to disseminate from the lungs to other tissues, which suggested that HBHA is essential for the infection of lung epithelial cells and extrapulmonary dissemination of *Mtb* (Pethe et al., 2001; Parra et al., 2004; Temmerman et al., 2005; Locht et al., 2006; Kohama et al., 2008).

Autophagy plays key roles in immune defense against invading pathogens. Autophagy also presents a protective role against *Mtb* infection (Kawamura, 2006; Rovetta et al., 2014). Thus, autophagy inhibition may facilitate *Mtb* infection. The present study is the first to report that HBHA protein could significantly inhibit LC3 expression and autophagosome formation in A549 cells, which indicated that autophagy could be suppressed by HBHA (Figures 1, 2). To further confirm this phenomenon, we constructed rHBHA protein-expressing *MS*. HBHA protein only exists in *Mtb* and *BCG* and not in *MS* (Zhao et al., 2012). Therefore, recombinant *MS* is an appropriate strain for observing HBHA protein function. The HBHA expression in *MS* exerted no effect on the growth of bacterium (Figure 4A), but significantly inhibited the expression of LC3 and maturation of autophagosome in A549 (Figure 3), eventually leading to attenuated clearance of *MS* by the cells. Consequently, the number and survival rate of intracellular bacteria increased significantly because of the reduced capacity to eliminate bacteria from infected cells (Figure 4B).

**Figure 7:**
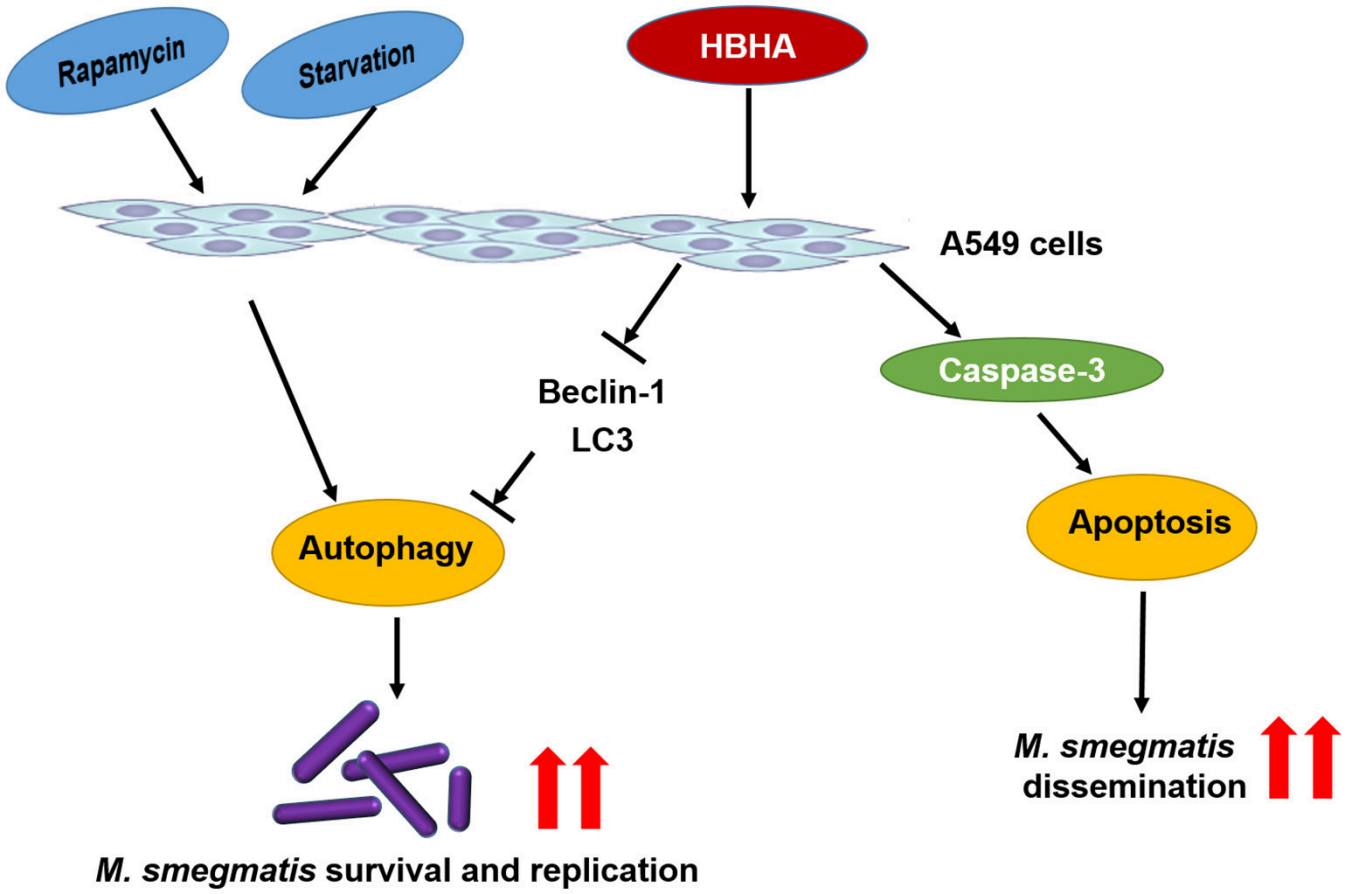
**Schematic model of the role of HBHA during mycobacterial infection**. HBHA inhibited autophagy in mycobacterial infected A549 cells, thereby promoted intracellular bacterial survival and replication. Subsequently, HBHA induced apoptosis on A549 cells through activation of caspase-3 which may facilitate bacterial escape from lung epithelial cells and dissemination to the adjacent cells.

Virulent *Mtb* induces necrosis of infected macrophages by inhibiting the repair process of the plasma membrane. This event leads to cellular lysis and reinforces spreading to adjacent infection sites (Chen et al., 2006, 2008; Divangahi et al., 2009). Recent reports suggested that a high intracellular burden of virulent *Mtb* induced macrophage cell death via a new apoptotic pathway involved in bacterial escape and extracellular replication (O'Sullivan et al., 2007). Similarly, our results demonstrated that *rMS-HBHA* could induce cell apoptosis of A549 cells through activation of caspase-3 (Figures 5, 6). This phenomenon may facilitate bacterial escape from lung epithelial cells and dissemination to the adjacent cells. The schematic model of the role of HBHA during mycobacterial infection was shown in Figure 7.

Induction of autophagy in macrophages is an effective mechanism to enhance intracellular killing of *Mtb*, and the ability of pathogen to inhibit this process is considerably important for its survival (Deretic et al., 2006; Songane et al., 2012). Beijing strains could resist autophagic killing by host cells compared with H37Rv and a strain belonging to the East African Indian genotype (Haque et al., 2015). The virulent strain H37Rv presents remarkably increased ability to inhibit autophagy flux than those of avirulent strains H37Ra and BCG, depending on virulence regulators PhoP, ESAT-6, and ESX-1 system, which controlled the secretin of ESAT-6 (Chandra et al., 2015). PE_PGRS47 (Rv2741) and ESAT-6/CFP10 are inhibitors of autophagosome formation in macrophages (Zhang et al., 2012; Saini et al., 2016). Nevertheless, *Mtb* used HBHA as a support when suppressing autophagy for survival and dissemination. However, whether HBHA can inhibit macrophage autophagy in macrophage and subsequently trigger apoptosis to allow bacterium to avoid macrophage killing should be further verified experimentally.

## Conclusion

Our study demonstrated that HBHA could inhibit autophagy of epithelial cells, facilitate *MS* intracellular survival, and promote its infection.

## Author contributions

QZhe, YM, and XH: Designed the experiments. QZhe, ZL, SZ, QZha, LZ, LY, and XF: Performed the experiments and analyzed the data. QZhe and YM: Wrote the paper. All authors reviewed the manuscript.

### Conflict of interest statement

The authors declare that the research was conducted in the absence of any commercial or financial relationships that could be construed as a potential conflict of interest.
